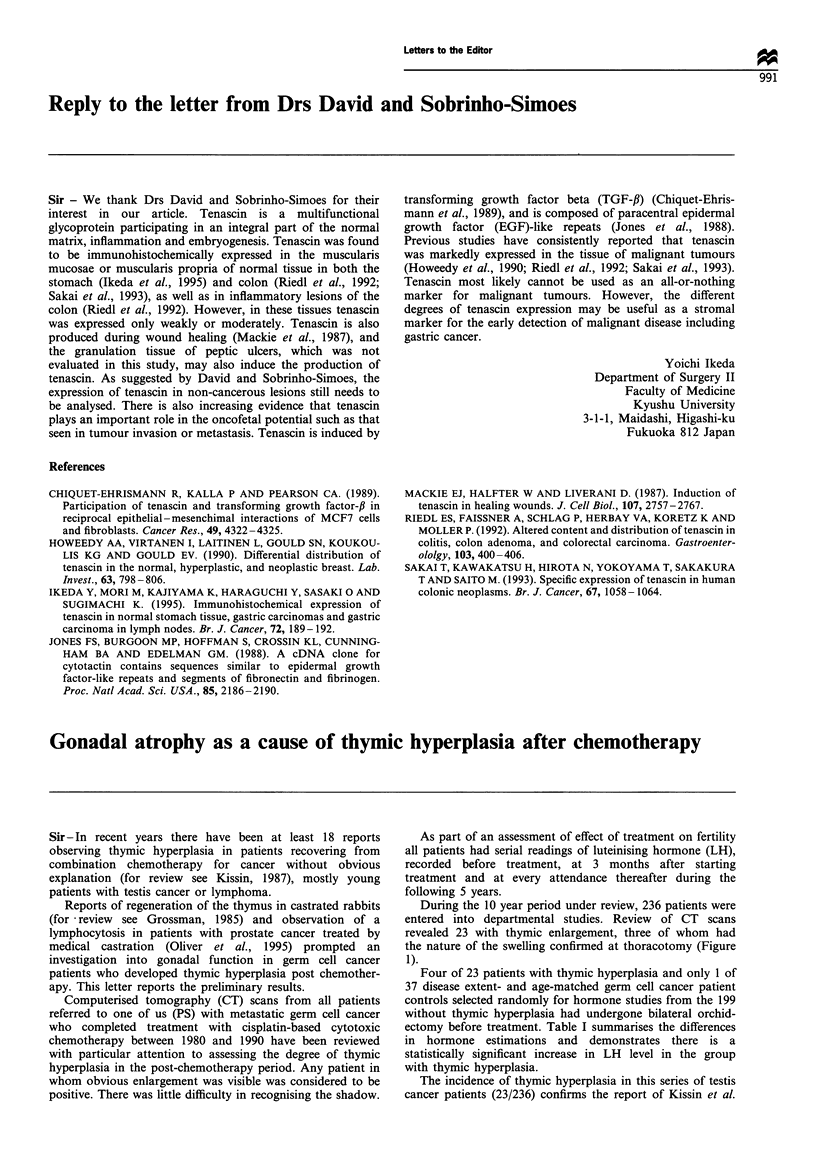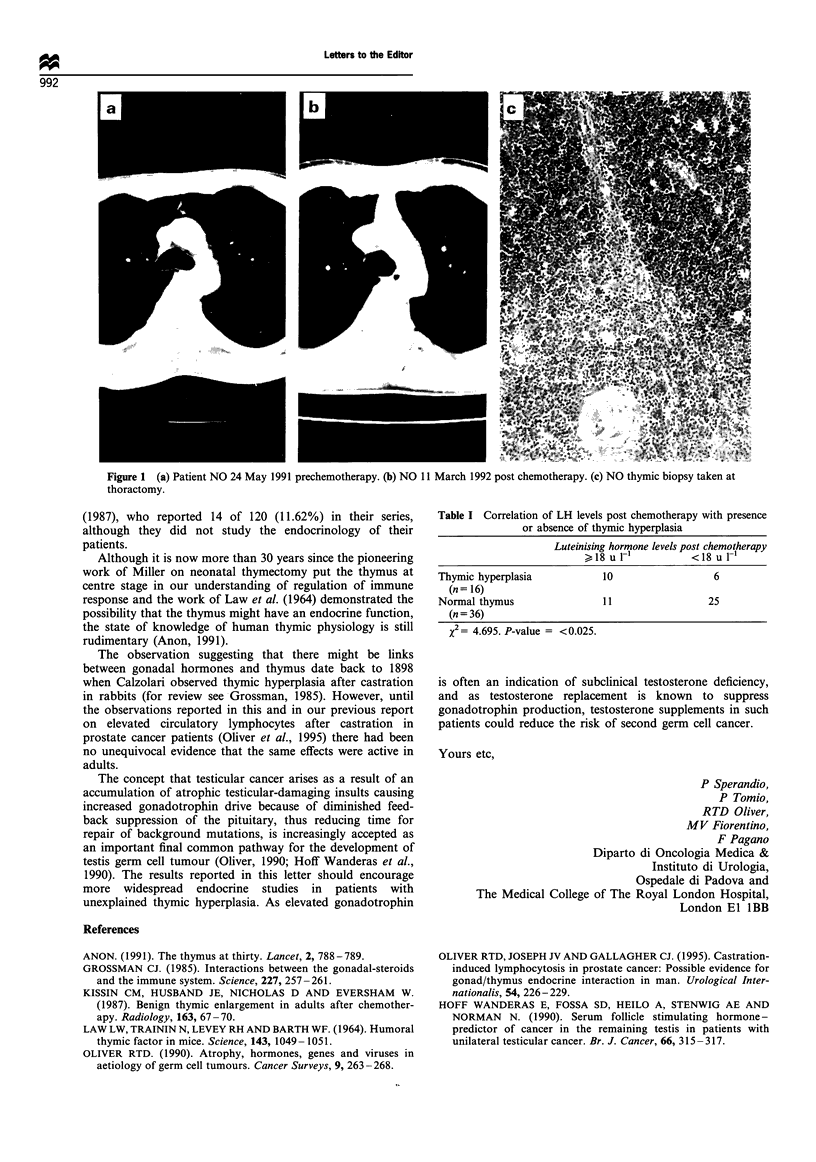# Gonadal atrophy as a cause of thymic hyperplasia after chemotherapy.

**DOI:** 10.1038/bjc.1996.474

**Published:** 1996-09

**Authors:** P. Sperandio, P. Tomio, R. T. Oliver, M. V. Fiorentino, F. Pagano

## Abstract

**Images:**


### Gonadal atrophy as a cause of thymic hyperplasia after chemotherapy

Sir – In recent years there have been at least 18 reports observing thymic hyperplasia in patients recovering from combination chemotherapy for cancer without obvious explanation (for review see Kissin, 1987), mostly young patients with testis cancer or lymphoma.

Reports of regeneration of the thymus in castrated rabbits (for review see Grossman, 1985) and observation of a lymphocytosis in patients with prostate cancer treated by medical castration (Oliver *et al.*, 1995) prompted an investigation into gonadal function in germ cell cancer patients who developed thymic hyperplasia post chemotherapy. This letter reports the preliminary results.

Computerised tomography (CT) scans from all patients referred to one of us (PS) with metastatic germ cell cancer who completed treatment with cisplatin-based cytotoxic chemotherapy between 1980 and 1990 have been reviewed with particular attention to assessing the degree of thymic hyperplasia in the post-chemotherapy period. Any patient in whom obvious enlargement was visible was considered to be positive. There was little difficulty in recognising the shadow.

As part of an assessment of effect of treatment on fertility all patients had serial readings of luteinising hormone (LH), recorded before treatment, at 3 months after starting treatment and at every attendance thereafter during the following 5 years.

During the 10 year period under review, 236 patients were entered into departmental studies. Review of CT scans revealed 23 with thymic enlargement, three of whom had the nature of the swelling confirmed at thoracotomy (Figure 1).

Four of 23 patients with thymic hyperplasia and only 1 of 37 disease extent- and age-matched germ cell cancer patient controls selected randomly for hormone studies from the 199 without thymic hyperplasia had undergone bilateral orchidectomy before treatment. Table I summarises the differences in hormone estimations and demonstrates there is a statistically significant increase in LH level in the group with thymic hyperplasia.

The incidence of thymic hyperplasia in this series of testis cancer patients (23/236) confirms the report of Kissin *et al.*

**Figure 1** (**a**) Patient NO 24 May 1991 prechemotherapy. (**b**) NO 11 March 1992 post chemotherapy. (**c**) NO thymic biopsy taken at thoracotomy.

(1987), who reported 14 of 120 (11.62%) in their series, although they did not study the endocrinology of their patients.

Although it is now more than 30 years since the pioneering work of Miller on neonatal thymectomy put the thymus at centre stage in our understanding of regulation of immune response and the work of Law *et al.* (1964) demonstrated the possibility that the thymus might have an endocrine function, the state of knowledge of human thymic physiology is still rudimentary (Anon, 1991).

The observation suggesting that there might be links between gonadal hormones and thymus date back to 1898 when Calzolari observed thymic hyperplasia after castration in rabbits (for review see Grossman, 1985). However, until the observations reported in this and in our previous report on elevated circulatory lymphocytes after castration in prostate cancer patients (Oliver *et al.*, 1995) there had been no unequivocal evidence that the same effects were active in adults.

The concept that testicular cancer arises as a result of an accumulation of atrophic testicular-damaging insults causing increased gonadotrophin drive because of diminished feedback suppression of the pituitary, thus reducing time for repair of background mutations, is increasingly accepted as an important final common pathway for the development of testis germ cell tumour (Oliver, 1990; Hoff Wanderas *et al.*, 1990). The results reported in this letter should encourage more widespread endocrine studies in patients with unexplained thymic hyperplasia. As elevated gonadotrophin is often an indication of subclinical testosterone deficiency, and as testosterone replacement is known to suppress gonadotrophin production, testosterone supplements in such patients could reduce the risk of second germ cell cancer.

**Table I** Correlation of LH levels post chemotherapy with presence or absence of thymic hyperplasia

| | *Luteinising hormone levels post chemotherapy* | |
|---|---|---|
| | $\geqslant 18\ u\ l^{-1}$ | $< 18\ u\ l^{-1}$ |
| Thymic hyperplasia ($n = 16$) | 10 | 6 |
| Normal thymus ($n = 36$) | 11 | 25 |

$\chi^2 = 4.695$. $P$-value $= < 0.025$.

Yours etc,

*P Sperandio,*
*P Tomio,*
*RTD Oliver,*
*MV Fiorentino,*
*F Pagano*
Diparto di Oncologia Medica &
Instituto di Urologia,
Ospedale di Padova and
The Medical College of The Royal London Hospital,
London E1 1BB

### References

ANON. (1991). The thymus at thirty. *Lancet*, **2,** 788–789.

GROSSMAN CJ. (1985). Interactions between the gonadal-steroids and the immune system. *Science*, **227,** 257–261.

KISSIN CM, HUSBAND JE, NICHOLAS D AND EVERSHAM W. (1987). Benign thymic enlargement in adults after chemotherapy. *Radiology*, **163,** 67–70.

LAW LW, TRAININ N, LEVEY RH AND BARTH WF. (1964). Humoral thymic factor in mice. *Science*, **143,** 1049–1051.

OLIVER RTD. (1990). Atrophy, hormones, genes and viruses in aetiology of germ cell tumours. *Cancer Surveys*, **9,** 263–268.

OLIVER RTD, JOSEPH JV AND GALLAGHER CJ. (1995). Castration-induced lymphocytosis in prostate cancer: Possible evidence for gonad/thymus endocrine interaction in man. *Urological Internationalis*, **54,** 226–229.

HOFF WANDERAS E, FOSSA SD, HEILO A, STENWIG AE AND NORMAN N. (1990). Serum follicle stimulating hormone–predictor of cancer in the remaining testis in patients with unilateral testicular cancer. *Br. J. Cancer*, **66,** 315–317.